# Haemolytic Anaemia-Related Pulmonary Hypertension

**DOI:** 10.3390/life14070876

**Published:** 2024-07-14

**Authors:** Panagiotis Karyofyllis, Eftychia Demerouti, Eleftheria-Garyfallia Tsetika, Styliani Apostolopoulou, Panagiotis Tsiapras, Ioannis Iakovou, Dimitrios Tsiapras

**Affiliations:** 1 Invasive Cardiology Department, Onassis Cardiac Surgery Center, 17674 Athens, Greece; pakar768@yahoo.gr (P.K.); elina_tse@live.com (E.-G.T.); ioannis.iakovou@gmail.com (I.I.); 2 Non-Invasive Cardiology Department, Onassis Cardiac Surgery Center, 17674 Athens, Greece; dtsiapras@hotmail.com; 3 School of Medicine, University of Thessaly, 41221 Larissa, Greece; sapostolopoulou@uth.gr; 4 Intensive Care Unit, Onassis Cardiac Surgery Center, 17674 Athens, Greece; pan.tsiapras@gmail.com

**Keywords:** pulmonary hypertension, haemolytic anaemia, haematological disorders, chronic thromboembolic pulmonary hypertension, pulmonary arterial hypertension, balloon pulmonary angioplasty

## Abstract

Haemolytic anaemia represents a risk factor for the development of pulmonary hypertension (PH), currently classified as World Health Organization group 5 PH, and data regarding appropriate therapeutic strategy are limited. A total of 28 patients, 85.7% with thalassaemia and 14.3% with sickle cell disease, with a diagnosis of PH confirmed by right heart catheterization were included in the study. The patients were divided into three groups according to the PH haemodynamic definition and overall diagnostic approach: 42.9% had precapillary PH (pulmonary arterial hypertension—PAH group), 25% had post-capillary PH, and 32.1% had chronic thromboembolic PH (CTEPH) (29% of b-thalassemia and 50% of SCD patients). The therapeutic approach in each group and its impact on the outcome and haemodynamics were recorded. PAH-specific drug therapy received 82.1% of patients, and balloon pulmonary angioplasty (BPA) was performed in six patients with CTEPH. There were statistically significant differences in baseline mPAP and PVR values between the CTEPH-haemolytic anaemia group and other groups. PAH-specific drug therapy resulted in haemodynamic improvement for the PAH group. Patients who underwent BPA had improved pulmonary haemodynamics. The median survival time was 162 months, and the survival rate was 1 year—100%; 2, 3, 4, 5, and 6 years—96%; 9 years—90%; and 13 years—78%. In patients with haemolytic anaemia, the wide spectrum of induced PH highlighted the importance of a correct predominant diagnosis. BPA in CTEPH patients and specific-PAH drug therapy for PAH patients represent potential therapeutic strategies; however, the management should be offered in expert PH centres under individualized approaches for patients.

## 1. Introduction

Hereditary haemoglobin disorders affecting the globin chain synthesis, thalassaemia syndromes, and sickle cell disease represent common genetic diseases, as around 7% of the world population carries genes for these disorders [1,2]. Pulmonary hypertension (PH) is a major complication due to increased pulmonary vascular resistance (PVR), leading to right ventricular dysfunction and right heart failure, representing a major cause of mortality and morbidity in chronic haemolytic anaemias [3,4]. PH is currently defined as an increase in mean pulmonary arterial pressure (mPAP) of more than 20 mmHg at rest, and is assessed by right heart catheterization (RHC); in all PH forms, pulmonary arterial wedge pressure (PAWP) is lower than 15 mmHg (precapillary PH), except in Group II PH due to left heart disease (PH-LHD), which is characterized by a PAWP of more than 15 mmHg (postcapillary PH) [5]. The pathogenesis of PH in haemolytic anaemia patients has not been fully clarified; however, accumulating evidence indicates that haemolysis, reduced nitric oxide bioavailability, increased endothelin-1-mediated responses, arginine metabolism dysregulation, compromised oxygenation, and chronic hypoxia, as well as hypercoagulability, play crucial roles in the development of the disease [6]. A variety of underlying mechanisms can lead to different forms of PH [7,8], such as pulmonary arterial hypertension (PAH), chronic thromboembolic PH (CTEPH), PH due to left heart disease (PH-LHD), and PH due to lung disease. Thus, haemolytic anaemias are included in group 5 PH according to the latest guidelines [5], which is the group defined as PH with unclear and multifactorial mechanisms. This classification does not permit a widely accepted standard therapeutic strategy for PH haemolytic anaemia patients, highlighting the importance of prompt diagnosis. Considering the unmet goal of the appropriate therapeutic approach for those patients, we retrospectively studied our haemolytic anaemia population.

## 2. Materials and Methods

### 2.1. Patient Selection

This single-centre, retrospective observational study was approved by the Institutional Review Board of Onassis Cardiac Surgery Centre (approval number 2020-686). The need for written informed consent was waived because of the retrospective nature of the study. Between January 2004 and April 2023, a total of 28 consecutive patients, 24 (85.7%) with β-thalassaemia major or intermedia and 4 patients (14.3%) with SCD who suffered from PH, were included. The diagnosis of PH was established by right heart catheterization according to the definition proposed in the 2022 guidelines for the diagnosis and treatment of PH. Haemodynamic and echocardiographic parameters were studied. The patients were divided in three groups based on haemodynamics and overall diagnostic approach according to the 2022 ESC/ERS guidelines [5]. All patients underwent lung ventilation perfusion scanning (V/P lung scanning) and additional invasive pulmonary angiography in the case of V/P lung scanning positivity, spiral chest computed tomography, spirometry, and all the diagnostic steps according to the recommendations in the guidelines.

**Figure 1:**
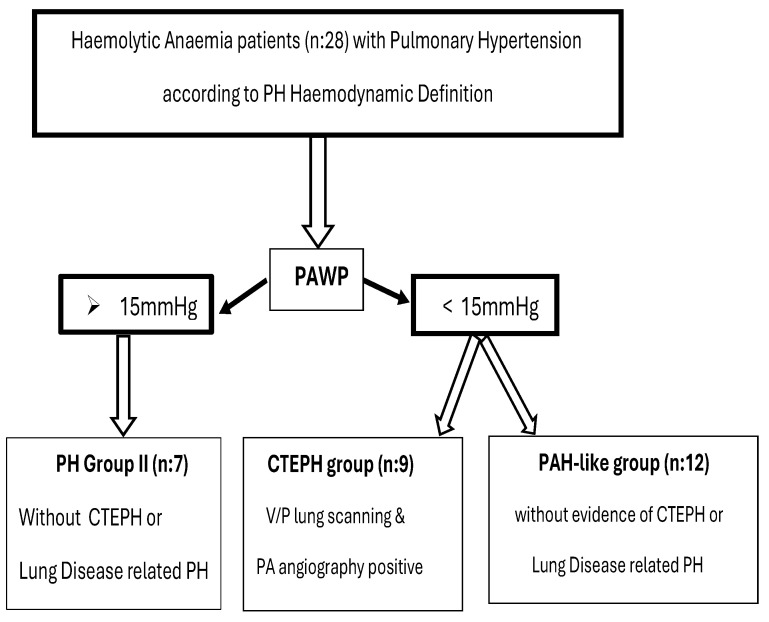
Study flowchart of participants, showing the whole group of patients and their allocation according to diagnostic approach. PH: pulmonary hypertension, PAWP: pulmonary arterial wedge pressure, CTEPH: chronic thromboembolic pulmonary hypertension, PA: invasive pulmonary angiography.

Seven patients (25%) with increased PAWP > 15 mmHg were classified as post-capillary PH patients (Group II PH). None of them had positive V/P lung scans or lung disease. Patients with PAWP < 15 mmHg were further classified as CTEPH patients (group IV-PH) when the V/P lung scan and pulmonary angiography were positive (9 patients—32.1%), and those with precapillary PH with no lung or thromboembolic disease were categorized as PAH-like group patients (12 patients, representing 42.9% of our cohort). The study flowchart of participants, showing the whole group of patients and their allocation according to diagnostic approach is shown in Figure 1.

### 2.2. Data Collection

Data concerning medical history, medication, and comorbidities were obtained from medical records. Echocardiographic parameters (right ventricular fractional area change (RVFAC), right atrial area (RAA), and tricuspid annular plane systolic excursion (TAPSE)/pulmonary arterial systolic pressure (PASP)) and hemodynamic parameters (mPAP, PASP, PAWP, right atrial pressure (RAP), cardiac output estimated by Fick method, and pulmonary vascular resistance (PVR)) were collected at baseline and at the last available follow-up visit.

Right-heart catheterization was performed at the beginning of each BPA procedure for CTEPH patients. For the post-treatment analysis of haemodynamics, the catheterization performed before the last BPA procedure was used, except for one patient for whom a follow-up catheterization after the end of treatment was available. BPA was performed in a series of staged procedures at several intervals depending on patients’ preferences. The target of BPA was to achieve an mPAP < 30 mmHg with concomitant cessation of oxygen therapy.

The follow-up period for analyses of survival data for the study population ended in October 2023.

### 2.3. Statistical Analysis

Data are presented as mean ± standard deviation for continuous variables with normal distribution, and as median and interquartile range for non-normally distributed variables. Differences in continuous variables before and after treatment were compared using the paired *t*-test for normally distributed variables and the Wilcoxon paired test for non-normally distributed variables. The normality was assessed with the Shapiro–Wilk test. Categorical variables are expressed as numbers and percentages and were compared using the χ2 test for independence or Fisher’s exact test. The Kruskal–Wallis test or one-way ANOVA with post hoc analysis were used as appropriate for comparisons between groups. Receiver operating characteristic analyses were used to determine the cut-off values for selected variables. The Kaplan–Meier method was used to estimate overall survival, and differences between survival curves were assessed using the log-rank test. For the survival analysis, the date of PH diagnosis was used as the start point to determine the length of survival. The cut-off date was October 2023. The Kaplan–Meier curve was supplemented by the proportion surviving at the analysed times. A *p*-value < 0.05 was considered statistically significant in this study. Data were analysed using SPSS version 16.0 (IBM SPSS Statistics for Windows, Version 16.0, Armonk, NY, USA).

## 3. Results

### 3.1. Baseline Haemodynamic and Echocardiographic Characteristics

The age at PH diagnosis was 48 ± 9 years (33–65), and 15 patients (53.6%) were females. Three patients had a history of pulmonary embolism, nine patients had atrial fibrillation, and three patients had diabetes mellitus. For the whole group, the mean pulmonary arterial pressure (mPAP) was 41.0 ± 9.8 mmHg, the PAWP was 13.2 ± 4.7 mmHg, the cardiac index (C.I.) was 2.7 ± 0.7 L/min/m^2^, the pulmonary vascular resistance (PVR) was 6.3 ± 3.0 WU, the venous oxygen saturation (SVO2) was 63.5 ± 9.8%, the pulmonary arterial compliance (PAC) was 1.69 ± 1.3, the right atrial pressure (RAP) was 10.4 ± 4.9 mmHg, and the systolic pulmonary arterial pressure (PASP) was 65.5 ± 17.7 mmHg. The diastolic pressure gradient is not mentioned in our analysis as its use is not further indicated for PH classification or risk stratification for PH patients.

Patients were also studied via echocardiography. The right ventricular fractional area (RVFAC) was 41.9 ± 4%, the tricuspid annulus plane systolic excursion (TAPSE)/PASP was 0.47 ± 0.16, and the right atrial area (RA area) was 16.1 ± 4 cm^2^. All included patients had normal left ventricular ejection fractions, without at least moderate left heart valvular disease or any type of cardiomyopathy.

Haemodynamic characteristics showed some significant differences among the three population groups (Table 1). The CTEPH group was more severely compromised, according to their haemodynamic parameters, than the two other groups in terms of PASP, mPAP, PVR, and SvO2 (*p* < 0.05).

CTEPH patients had more severe PH compared to patients with PAH, as mPAP was 50.4 ± 6.2 vs. 36 ± 7.6 mmHg (*p*: 0.01), PVR was 8.5 ± 2.4 vs. 5.7 ± 3 WU (*p* = 0.038), and SVO2 was 52.5 ± 10% vs. 68.3 ± 5.9% (*p* < 0.01) (Table 2). Apart from the haemodynamic parameters, the burden of CTEPH patients is also reflected in echocardiographic indices of right ventricular function, such as right ventricular fractional area change (RVFAC) and tricuspid annulus plane systolic excursion (TAPSE)/PASP, parameters which are significantly lower compared to patients with precapillary PH (RVFAC: 30 ± 2.5 vs. 42.8 ± 5%, *p* < 0.01 and TAPSE/SPAP: 0.27 ± 0.05 vs. 0.5 ± 0.17, *p* = 0.01, respectively). RVFAC was also significantly lower in CTEPH patients compared to the postcapillary group, Group II PH (38.3 ± 3.8%, *p* = 0.013), while no differences were noticed in these indices between pre- and postcapillary patients. Patients with precapillary PH classified into the PAH group had significantly lower RAP of 7.7 ± 4 vs. 13.5 ± 3.7 mmHg (*p* = 0.029) and PAWP of 10.8 ± 4 vs. 19 ± 1 mmHg (<0.01) compared to patients with postcapillary PH. No differences were noted in other haemodynamic parameters between these two groups. It is also important to note that six patients with PH-LHD had combined post- and precapillary PH.

### 3.2. Therapeutic Approach

PAH-specific drug therapy was prescribed to 23 (82.1%) patients (Table 2). Endothelin receptor antagonist (ERA) was prescribed to 15 (65.2%) patients (ambrisentan in 39.1%, bosentan in 17.4%, and macitentan in 8.7%), Phosphodiesterase-5 inhibitors (PDE-5) were prescribed to six (26%) patients and Riociguat to eight (34.5%) of patients. Considering the SCD group, three patients received Riociguat and a patient combination therapy with ERA and Selexipag. Sixteen patients received monotherapy and seven patients received double PAH drug therapy. One patient with precapillary PH did not receive drug therapy as mild PH was identified (mPAP 26 mmHg). The majority of patients with postcapillary PH (57.1%) did not receive specific PAH drugs.

### 3.3. Hemodynamic Parameters at Follow-Up

#### 3.3.1. PAH-like Group

Nine out of twelve PAH group patients had available baseline and post-treatment haemodynamic parameters. An improvement was noted in PASP, PVR, and PAC for patients who received PAH-specific drugs (Table 3). Furthermore, in 10 patients studied by echocardiography before and after treatment, a significant improvement in TAPSE/PASP was noticed (0.47 ± 0.16 vs. 0.69 ± 0.22, *p* = 0.039).

#### 3.3.2. PH Group II (PH-LHD)

Five out of seven patients with postcapillary PH had follow-up catheterization, and among all aforementioned haemodynamic parameters, only RAP was significantly improved (14.2 ± 3.3 vs. 9.4 ± 2.0 mmHg, *p* = 0.026) (Table 4). Three patients received PAH-specific drug therapy. Patients 1, 2, and 3 had mPAP 39 mmHg, 32 mmHg, and 56 mmHg, respectively, and PVR 3.1, 4.3, and 7.9 WU, respectively. The rest of the patients had PVR < 4.1 WU. No difference was noted between patients who received PAH-specific drug therapy and those who did not receive it. Patient No. 3, with the most severe haemodynamically defined PH, had a PVR which improved to 3.9 WU and a mPAP which improved to 37 mmHg after drug therapy.

#### 3.3.3. CTEPH Group

CTEPH patients had improved PASP, mPAP, and PVR after treatment. Balloon pulmonary angioplasty (BPA) was performed in six (66.7%) of nine patients with CTEPH, as all of them had distal disease; all CTEPH patients were receiving specific PAH drug therapy. BPA in Greece has been available since 2017. One patient among the three who did not undergo BPA died before that year; the second was diagnosed 11 years before starting BPA program and died two years after the initiation of the program without consenting to undergo evaluation for BPA eligibility; and the third one was considered ineligible due to extremely severe disease with very hard lesions extending to distal vasculature and an unfavourable risk-to-benefit ratio. Baseline and follow-up haemodynamic data were available for patients who underwent BPA (Table 5).

The improvement in haemodynamics for patients undergoing BPA is anticipated to be more pronounced if the treatment were continued to reach treatment goals. Among these six patients, one patient died after an accident and subsequent pulmonary embolism before completion of therapy and after only two sessions. Two other patients did not consent to the continuation of interventions due to significant clinical improvement despite not reaching the target of mPAP < 30 mmHg (both with 32 mmHg before the last BPA). One patient became normal (mPAP from 56 to 18 mmHg), and the last two patients continued interventions.

#### 3.3.4. Haemodynamic Indices to Predict the Relevant PH Diagnosis

All haemodynamic indices were further analysed for their ability to predict the PH group that the patients were ultimately included in, and especially to differentiate CTEPH from the other two groups, using receiver operating characteristics (ROC) curves with corresponding area under the ROC curves (AUCs). mPAP had the highest AUC to predict CTEPH diagnosis (0.895, *p*: 0.001), and PVR had an AUC of 0.833 (*p*: 0.0050). The optimal cut-off values for mPAP and PVR were 42 mmHg, with a sensitivity of 100% and specificity of 79%, and 5.3 WU, with a sensitivity of 89% and specificity of 74%, in predicting CTEPH diagnosis, respectively.

### 3.4. Survival

**Figure 2:**
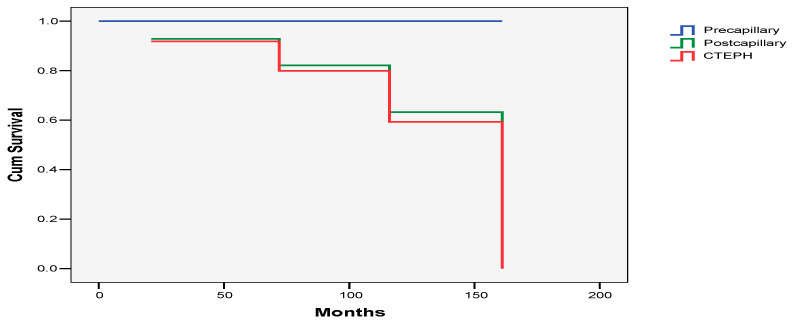
Overall survival according to the different PH groups in the study population.

Overall survival at 1 year was 100%; that at 2, 3, 4, 5, and 6 years was 96%; that at 9 years was 96%; and that at 13 years was 78%. The median survival time was 162 months (Figure 2). From the whole study group, four patients died, one with postcapillary PH and the other three with CTEPH. The survival rates between patients with postcapillary PH and CTEPH were similar (*p* = 0.916), but there was a trend of the worst survival rate in patients with postcapillary PH and CTEPH as compared with patients with precapillary PH (*p* = 0.068 and *p* = 0.061, respectively).

## 4. Discussion

PH associated with haemolytic anaemia was classified as Group I PH (PAH) during the 4th PH World Congress; however, at the 2013 5th World Symposium on PH, reclassification to Group V PH was proposed by the Task Force due to the multifactorial pathogenesis of haemolytic anaemia resulting in different PH forms. Chronic haemolysis leads to free haemoglobin, resulting in inactivation and reduced NO synthesis, as well as alteration of the L-arginine metabolism, contributing to smooth muscle proliferation and vascular remodelling. Furthermore, haemolytic disorders increase plasma endothelin-1 levels, leading to vasoconstriction. These proposed mechanisms represent disorders underlying the development of precapillary PH [9,10]. Moreover, red cell destruction can provoke platelet and tissue factor activation and thrombin generation, leading to obliteration. Splenectomy, endothelial dysfunction, and red cell pre-coagulant surface are considered as causes of thromboembolic complications resulting in thromboembolic disease and, subsequently, PH. Iron overload and high cardiac output state are major causes of left heart dysfunction, so patients with haemolytic anaemia can ultimately develop PH due to left heart disease [10,11]. Iron overload, mostly present in transfusion-dependent patients with b-thalassaemia, may induce interstitial pulmonary fibrosis leading to PH due to lung disease (Group III PH) [12]. A combination of these abnormalities in several patients can lead to mixed PH cases; however, right heart catheterization is mandatory to distinguish precapillary and postcapillary PH. Echocardiography does not seem to provide accurate pulmonary pressures in haemolytic anaemia cohorts, according to the literature [13]. In our cohort, all symptomatic haemolytic anaemia patients with intermediate and high echocardiographic probability for PH underwent RHC. They were classified into three groups as follows: group II PH (PH-LHD) and those with precapillary PH subsequently devised in CTEPH patients and PAH-like group patients. None of our patients had severe parenchymal disease according to high-resolution chest computed tomography, pulmonary function tests, or blood gas analysis, which are mandatory steps in the diagnostic PH algorithm to exclude PH due to a lung disease diagnosis.

According to the literature, thrombotic lesions are a major component of PH related to SCD [2], and precapillary PH has an important impact on survival, with an overall mortality rate of 5.3% in 18 months [1]. In b-thalassemia, precapillary PH appears in 2.1% of cases, and postcapillary in 0.3% [4]. Among our patients with thalassemia, 45.6% had the PAH phenotype, 25% had postcapillary PH, and 29.4% had CTEPH. Based on haemodynamic characteristics, CTEPH patients are more severely compromised compared to the other two groups. Furthermore, the levels of PVR and mPAP are identified as indices to predict CTEPH diagnosis. This finding could differentiate diagnosis between precapillary PH with thromboembolic disease versus that without. Thus, during a diagnostic approach, values of mPAP or PVR of more than 42 mmHg and 5.3 WU, respectively, necessitate CTEPH exclusion via a lung V/Q scan. If the V/Q scan is positive, invasive pulmonary angiography is a cornerstone for CTEPH verification, emerging as an assessment for a possible invasive therapeutic approach and ameliorating patients’ outcomes.

Prompt PH classification is mandatory for the most appropriate therapeutic approach. There are no specific management guidelines for patients with PH associated with haemolytic anaemia [5]; however, it is of paramount importance for them to be treated with oxygen supplementation when hypoxia is present, as well as with blood transfusion and iron chelation according to the severity of underlying haemolytic anaemia disease. Specific PAH drugs, including ERAs, PDE5, Riociguat, prostacyclin receptor agonists such as Selexipag, and prostanoids, have been studied in a small number of patients, and there is a lack of data supporting their use in haemolytic anaemia patients [14]. PDE-5 was tried in few small case series, with positive results concerning 6-minute walk distance, NYHA functional class, and decreased tricuspid regurgitation velocity [14,15]. A multi-centre, randomized, double-blind trial [15] (walk-PHaSST trial) was terminated early because of increased hospitalization rates due to pain for SCD patients treated with Sildenafil. This is the reason that PDE-5 has been avoided in this cohort. Aclinical trial with fourteen SCD patients treated with ERAs showed improvement in mPAP [16]. Acute administration of intravenous epoprostenol in patients with SCD-PH has shown a decrease in PVR and an increase in cardiac output, resulting in a worsening hyperdynamic state and heart failure [14]. Our study reveals haemodynamic improvement for patients treated with PAH-specific drug therapy when classified as precapillary PH. Careful PAH drug initiation with regular follow-up and re-catheterization in 3–6 months is appropriate after specific PAH drug therapy initiation.

Furthermore, the effectiveness of specific PAH drugs in these patients is also reflected in the excellent survival they showed in contrast to the survival of patients suffering from several forms of group 1 PH, such as patients with idiopathic PAH or PAH associated with connective tissue disorders. Castro et al. demonstrated a median survival for SCD patients suffering from PH 25, 6 months in a 119-month observation period [17]. In our cohort, the precapillary group had 100% survival for more than 12 years, while the 7-year survival in the drug era for PAH patients is only 49% according to the REVEAL registry [18]. The good survival for chronic haemolytic anaemia patients classified as suffering with PAH in our cohort differs substantially from general-population PAH patients included in registries. The reasons are not well established; however, the compensatory high-cardiac-output-state haemolytic anaemia patients have lower pulmonary resistance with comparable levels of pressure. Furthermore, managing tissue hypoxia via the intensification of blood transfusion and chelation therapy, the use of hydroxyurea, and hypercoagulable status improvement seem to be effective therapeutic tools in ameliorating patients’ status and prognosis. Our results are not also comparable with a previous report (11) that shows worse PAH prognosis in thalassaemic patients. Following diagnosis, all patients in our cohort were transfused with Hb levels >10.0 g/dL, and the chelation therapy was based on mean ferritin levels <300 mg/dL. This is different than study #11. Moreover, in this study design [4,11], exclusion of CTEPH was performed by computed tomography pulmonary angiography (CTPA) and not by V/Q lung scan, as suggested by established guidelines [5,6]. The diagnostic accuracy of CTPA in CTEPH is limited [19]. In our study, all patients with abnormal V/Q lung scans underwent invasive digital pulmonary angiography, showing predominantly distal lesions, a finding which is not easily identified in CTPA even if it is present and which is associated with a worse prognosis. BPA in distal thrombotic lesions in the pulmonary vascular bed in our cohort resulted in better outcomes.

PAH-specific drug therapy is further recommended in CTEPH patients, as micro-vasculopathy is implicated in pathogenesis. Riociguat and Treprostinil are indicated according to RCTs [20,21]; however, to manage the increased PVR, medical therapies are usually used off-label according to uncontrolled studies and/or regional approvals [5]. CTEPH management includes a multimodal approach of a combination of pulmonary endarterectomy (PEA), BPA, and medical therapies, and all patients should be evaluated for possible pulmonary endarterectomy [5]. PEA represents the gold standard of treatment, and BPA remains the alternative option for those deemed to be inoperable or when thrombi do not appear in the main or segmental pulmonary arterial branches. Data from the RACE trial [22] demonstrated that, for patients who underwent BPA, pre-treatment with Riociguat was associated with a lower complication rate (14% vs. 42% of patients). There is not much data on PEA or BPA in chronic haemolytic anaemia [23,24,25,26]. According to Papworth’s experience, 19 patients with haemoglobinopathy of congenital haemolytic anaemia underwent PEA [27]. The results of PEA in this complex patient group were satisfactory under expert haematological advice and exchange blood transfusions. Immediately postoperatively, there was a significant improvement in PVR, so the researchers concluded that the presence of abnormal haemoglobin does not contraindicate PEA surgery. In our cohort, the thromboembolic disease was distal in the pulmonary vascular bed, so BPA seemed to be the proper therapeutic management, and CTEPH patients were treated with PAH-specific drug therapy and most of them with BPA. Haemodynamic improvement was achieved with a survival of 60% in 162 months.

### Limitations of the Study

Although it is limited by a small sample size, our study highlights the importance of prompt PH classification and furthers our knowledge on appropriate therapeutic management based on the prompt diagnosis. Data on the benefits of PAH-specific drug therapy and of BPA are limited due to a low number of small clinical trials or registries, and although this study was not conducted to evaluate their impact on the mortality, haemodynamic improvements are present and indicate the necessity of future randomized trials. The retrospective nature of the study is also a limitation; however, a retrospective research can be crucial when it allows the spread of scientific knowledge to have an impact on unknown or unclear scientific fields or when it offers a stimulus to design prospective studies [28]. Moreover, this single-centre cohort study was performed in an expert PH centre, and it is well established that for small sample sizes, single-centre discovery sets show superior performance with respect to the chance of successful validation [29]. Furthermore, it is of great importance that the study is based on haemodynamic parameters, as most of the published manuscripts on PH due to haemolytic anaemia are based on echocardiographic indices.

## 5. Conclusions

PH is a complication in chronic haemolytic anaemia patients, and its multifactorial pathogenesis is responsible for multiple phenotypes, which are ultimately classified into different PH groups. The heterogeneity of patient phenotypes and the wide spectrum of disease necessitate a prompt diagnosis based on right heart catheterization and the whole diagnostic approach, according to the recommendations of current guidelines. It is of utmost importance to classify haemolytic anaemia patients into the most appropriate PH Group, highlighting the possible predominant underlying pathophysiologic mechanism, the cornerstone for the most appropriate therapeutic approach. We propose initial CTEPH exclusion via V/P lung scintigraphy and, in case of positivity, verification by invasive pulmonary angiography. Furthermore, if postcapillary PH is excluded, the haemolytic anaemia patient should be classified as a PAH-like group patient. Lung disease may contribute to PH development; however, when thromboembolic material is present in the pulmonary vascular bed, the patient may be treated invasively, as guidelines for CTEPH management suggest. During the diagnostic approach, values of PVR and mPAP of more than 5.3 WU and 42 mmHg, respectively, can represent red flag signs, indicating the need for further CTEPH exclusion, as a sensitivity of 100% and specificity of 79% for the mPAP, and 89% and 74%, respectively, for the PVR, was found when predicting CTEPH diagnosis.

The role of PAH-specific drug treatment is not well established; however, prompt evaluation related to a correct diagnosis and classification could lead to better therapeutic management. Chronic thromboembolic phenotype patients seem to be the most severe population according to haemodynamic parameters. Although CTEPH haemolytic anaemia patients are more severely compromised, the improvement in haemodynamics for patients undergoing BPA seem to be more pronounced than the rest of the population. Thus, the prompt therapeutic approach for haemolytic anaemia patients can be described as follows: CTEPH patients can be managed invasively, and additional specific PH drug therapy can be prescribed when lung disease or postcapillary PH is not present. Specific PAH drug therapy should be avoided in PH group II patients (PH-LHD) according to the guidelines, and PAH-like group patients can be treated with PAH-specific drug therapy in the absence of a diagnosis of at least moderate lung disease.

Given the complexity of haemolytic anaemia and PH therapy, as well as the rapidly changing landscape, these patients should be monitored and regularly receive follow-up in thalassaemia and PH expert centres. More information in future studies with long-term patient follow-up and outcomes could provide additional insights into the effectiveness and sustainability of the therapeutic strategies to be applied in this specific population.

## Figures and Tables

**Table 1:** Haemodynamic and echocardiographic parameters in the three study population groups.

	PAH Group (Group 1, n = 12)	PH-LHD (Group 2, n = 7)	CTEPH (Group 4, n = 9)	*p*-Value
RAP (mmHg)	7.7 ± 0.4	13.5 ± 3.7	11.5 ± 5.3	0.029 (group 1 vs. 2)
PASP (mmHg)	57.6 ± 12.8	55.8 ± 15.5	83.6 ± 10.5	≤0.01 (group 4 vs. 1, 2)
mPAP (mmHg)	36 ± 7.6	37.1 ± 8.6	50.4 ± 6.2	≤0.04 (group 4 vs. 1, 2)
PAWP (mmHg)	10.8 ± 4	19 ± 1.4	11.9 ± 3.2	<0.01 (group 2 vs. 1, 4)
CI (L/min/m^2^)	2.82 ± 0.78	2.67 ± 0.62	2.60 ± 0.7	ns
PVR (WU)	5.7 ± 3	4.3 ± 1.9	8.5 ± 2.4	≤0.038 (group 4 vs. 1, 2)
PAC (mL/mmHg)	1.78 ± 0.68	2.31 ± 2.36	1.09 ± 0.39	0.023 (group 2 vs. 4)
SVO2 (%)	68.3 ± 5.9	66.4 ± 6.1	52.5 ± 10	≤0.01(group 4 vs. 1, 2)
RVFAC (%)	42.8 ± 5.0	38.3 ± 3.7	30.0 ± 2.5	≤0.013 (group 4 vs. 1, 2)
TAPSE/PASP	0.50 ± 0.17	0.42 ± 0.19	0.27 ± 0.05	0.010 (group 4 vs. 1)
RA area (cm^2^)	16.5 ± 4.0	18 ± 4.3	21.0 ± 7.0	ns

PAH: pulmonary arterial hypertension, CTEPH: chronic thromboembolic pulmonary hypertension, PH-LHD: left heart disease-related pulmonary hypertension, RAP: right atrial pressure, PASP: systolic pulmonary arterial pressure, mPAP: mean pulmonary arterial pressure, PAWP: pulmonary arterial wedge pressure, ns: non significant PVR: pulmonary vascular resistance, PAC: pulmonary arterial compliance, SVO2: venous oxygen saturation, RVFAC: right ventricular fractional area, TAPSE: tricuspid annulus plane systolic excursion, RA area: right atrial area.

**Table 2:** Specific PH treatment among the three different groups of PH in the study patients.

Specific PH Treatment n = 23/28 (82.1)	ERA 15 (65.2)	PDE5 6 (26)	RIO 8 (34.5)	SELEX 1 (4.3)	Monotherapy 16 (69.5)	Double Therapy 7 (30.4)	No Specific PH Treatment 5 (17.9)
Precapillary PH n = 11/12	9 (75)	3 (25)	1 (8.3)	0	9 (75)	2 (16.7)	1 (8.3)
Postcapillary PH n = 3/7	1 (14.3)	1 (14.3)	2 (28.6)	0	2 (28.6)	1 (14.3)	4 (57.1)
CTEPH n = 9/9	5 (55.6)	2 (22.2)	5 (55.6)	1 (11.1)	5 (55.6)	4 (44.4)	0

PH: pulmonary hypertension, ERA: endothelin receptor antagonist, PDE5: phosphodiesterase inhibitor 5, RIO: Riociguat, SELEX: Selexipag, CTEPH: chronic thromboembolic pulmonary hypertension.

**Table 3:** RHC parameters at baseline and after PAH-specific drug treatment in PAH-like group.

n = 9	Baseline	Post Treatment	*p*-Value
RAP (mmHg)	8.1 ± 4.6	7.6 ± 2.8	0.807
PASP (mmHg)	62.6 ± 10.1	51 ± 9.6	0.019
mPAP (mmHg)	38.8 ± 6.8	33 ± 6.5	0.083
CI (L/min/m^2^)	2.92 ± 0.87	3.49 ± 0.91	0.145
PVR (WU)	6.4 ± 3.2	4 ± 1.9	0.005
PAC (mL/mmHg)	1.58 ± 0.55	2.75 ± 1.67	0.044
SVO2 (%)	67.5 ± 6	71.4 ± 6.5	0.094

RAP: right atrial pressure, PASP: systolic pulmonary arterial pressure, mPAP: mean pulmonary arterial pressure, CI: cardiac index, PVR: pulmonary vascular resistance, PAC: pulmonary arterial compliance, SVO2: venous oxygen saturation, WU: Woods units.

**Table 4:** Right heart catheterization parameters at baseline and at follow-up in postcapillary PH group.

	Baseline	Last RHC	*p*-Value
RAP (mmHg)	14.2 ± 3.3	9.4 ± 2	0.026
PASP (mmHg)	56.8 ± 18.9	55 ± 11	0.841
mPAP (mmHg)	38 ± 10.1	34.6 ± 5.5	0.482
CI (L/min/m^2^)	2.85 ± 0.64	2.76 ± 0.75	0.821
PVR (WU)	4.1 ± 2.3	4.2 ± 1.5	0.983
PAC (mL/mmHg)	2.71 ± 2.77	2.01 ± 0.78	0.594
SVO2 (%)	69.8 ± 1.2	66.1 ± 4.3	0.159

RAP: right atrial pressure, PASP: systolic pulmonary arterial pressure, mPAP: mean pulmonary arterial pressure, CI: cardiac index, PVR: pulmonary vascular resistance, PAC: pulmonary arterial compliance, SVO2: venous oxygen saturation.

**Table 5:** Haemodynamic parameters at baseline and after therapeutic approach in CTEPH patients.

n = 6	Baseline	Post Treatment	*p*-Value
RAP (mmHg)	11.5 ± 5.3	7.1 ± 1.4	0.149
PASP (mmHg)	85.2 ± 7.4	56.3 ± 21	0.02
mPAP (mmHg)	51.7 ± 6.1	34 ± 10.2	0.029
CI (L/min)	2.33 ± 0.35	2.79 ± 0.42	0.063
PVR (WU)	9.5 ± 1.6	4.5 ± 2.9	0.020
PAC (mL/mmHg)	0.94 ± 0.18	2.51 ± 2	0.122
SVO2 (%)	51.6 ± 10.9	61.1 ± 8.4	0.138

RAP: right atrial pressure, PASP: systolic pulmonary arterial pressure, mPAP: mean pulmonary arterial pressure, CI: cardiac index, PVR: pulmonary vascular resistance, PAC: pulmonary arterial compliance, SVO2: venous oxygen saturation.

## Data Availability

The data presented in this study are available on request from the corresponding author.

## References

[1] Gladwin M.T., Sachdev V., Jison M.L., Shizukuda Y., Plehn J.F., Minter K., Brown B., Coles W.A., Nichols J.S., Ernst I. (2004). Pulmonary hypertension as a risk factor for death in patients with sickle cell disease. N. Engl. J. Med..

[2] Savale L., Habibi A., Lionnet F., Maitre B., Cottin V., Jais X., Chaouat A., Artaud-Macari E., Canuet M., Prevot G. (2019). Clinical phenotypes and outcomes of precapillary pulmonary hypertension of sickle cell disease. Eur. Respir. J..

[3] Sutton L.L., Castro O., Cross D.J., Spencer J.E., Lewis J.F. (1994). Pulmonary hypertension in sickle cell disease. Am. J. Cardiol..

[4] Derchi G., Galanello R., Bina P., Cappellini M.D., Piga A., Lai M.E., Quarta A., Casu G., Perrotta S. (2014). Prevalence and risk factors for pulmonary arterial hypertension in a large group of beta-thalassemia patients using right heart catheterization: A Webthal study. Circulation.

[5] Humbert M., Kovacs G., Hoeper M.M., Badagliacca R., Berger R.M.F., Brida M., Carlsen J., Coats A.J.S., Escribano-Subias P., Ferrari P. (2022). 2022 ESC/ERS Guidelines for the diagnosis and treatment of pulmonary hypertension. Eur. Heart J..

[6] Galiè N., Humbert M., Vachiery J.L., Gibbs S., Lang I., Torbicki A., Simonneau G., Peacock A., Vonk Noordegraaf A., Beghetti M. (2016). 2015 ESC/ERS Guidelines for the diagnosis and treatment of pulmonary hypertension: The Joint Task Force for the Diagnosis and Treatment of Pulmonary Hypertension of the European Society of Cardiology (ESC) and the European Respiratory Society (ERS): Endorsed by: Association for European Paediatric and Congenital Cardiology (AEPC), International Society for Heart and Lung Transplantation (ISHLT). Eur. Heart J..

[7] Kremastinos D.T., Tsiapras D.P., Tsetsos G.A., Rentoukas E.I., Vretou H.P., Toutouzas P.K. (1993). Left ventricular diastolic Doppler characteristics inbeta-thalassemia major. Circulation.

[8] Aessopos A., Stamatelos G., Skoumas V., Vassilopoulos G., Mantzourani M., Loukopoulos D. (1995). Pulmonary hypertension and right heart failure inpatients with beta-thalassemia intermedia. Chest.

[9] Anthi A., Orfanos S.E., Armaganidis A. (2013). Pulmonary hypertension in β thalassaemia. Lancet Respir. Med..

[10] Mehari A., Klings E.S. (2016). Chronic Pulmonary Complications of Sickle Cell Disease. Chest.

[11] Pinto V.M., Musallam K.M., Derchi G., Graziadei G., Giuditta M., Origa R., Barella S., Casu G., Pasanisi A., Longo F. (2022). Mortality in β-thalassemia patients with confirmed pulmonary arterial hypertension on right heart catheterization. Blood.

[12] Parent F., Bachir D., Inamo J., Lionnet F., Driss F., Loko G., Habibi A., Bennani S., Savale L., Adnot S. (2011). A hemodynamic study of pulmonary hypertension in sickle cell disease. N. Engl. J. Med..

[13] Anthi A., Tsiapras D., Karyofyllis P., Voudris V., Armaganidis A., Orfanos S. (2021). The wide spectrum of β-thalassaemia intermedia-induced pulmonary hypertension: Two case reportson the possible role of specific pulmonary arteri-alhypertension therapy. Pulm. Circ..

[14] Derchi G., Forni G.L., Formisano F., Cappellini M.D., Galanello R., D’Ascola G., Bina P., Magnano C., Lamagna M. (2005). Efficacy and safety of sildenafil in the treatment of severe pulmonary hypertension in patients with hemoglobinopathies. Haematologica.

[15] Machado R.F., Martyr S., Kato G.J., Barst R.J., Anthi A., Robinson M.R., Hunter L., Coles W. (2005). Sildenafi l therapy in patients with sickle cell disease and pulmonary hypertension. Br. J. Haematol..

[16] Minniti C.P., Machado R.F., Coles W.A., Sachdev V., Gladwin M.T., Kato G.J. (2009). Endothelin receptor antagonists for pulmonary hypertension in adult patients with sickle cell disease. Br. J. Haematol..

[17] Castro O., Hoque M., Brown B.D. (2003). Pulmonary hypertension in sickle cell disease: Cardiac catheterization results and survival. Blood.

[18] Benza R.L., Miller D.P., Barst R.J., Badesch D.B., Frost A.E., McGoon M.D. (2012). An evaluation of long-term survival from time of diagnosis in pulmonary arterial hypertension from the REVEAL Registry. Chest.

[19] Dong C., Zhou M., Liu D., Long X., Guo T., Kong X. (2015). Diagnostic accuracy of computed tomography for chronic thromboembolic pulmonary hypertension: A systematic review and meta-analysis. PLoS ONE.

[20] Ghofrani H.A., D’Armini A.M., Grimminger F., Hoeper M.M., Jansa P., Kim N.H., Mayer E., Simonneau G., Wilkins M.R., Fritsch A. (2013). Riociguat for the treatment of chronic thromboembolic pulmonary hypertension. N. Engl. J. Med..

[21] Sadushi-Kolici R., Jansa P., Kopec G., Torbicki A., Skoro-Sajer N., Campean I.A., Halank M., Simkova I., Karlocai K., Steringer-Mascherbauer R. (2019). Subcutaneous treprostinil for the treatment of severe non-operable chronic thromboembolic pulmonary hypertension (CTEPH): A double-blind, phase 3, randomised controlled trial. Lancet Respir. Med..

[22] Jaïs X., Brenot P., Bouvaist H., Jevnikar M., Canuet M., Chabanne C., Chaouat A., Cottin V., De Groote P., Favrolt N. (2022). Balloon pulmonary angioplasty versus riociguat for the treatment of inoperable chronic thromboembolic pulmonary hypertension (RACE): A multicentre, phase 3, open-label, randomised controlled trial and ancillary follow-up study. Lancet Respir. Med..

[23] Karyofyllis P., Tsiapras D., Papadopoulou V., Diamantidis M.D., Fotiou P., Demerouti E., Voudris V. (2018). Balloon pulmonary angioplasty is a promising option in thalassemic patients with inoperable chronic thromboembolic pulmonary hypertension. Case Rep. J. Thromb. Thrombolysis.

[24] Demerouti E., Karyofyllis P., Voudris V., Boutsikou M., Anastasiadis G., Anthi A., Arvanitaki A., Athanassopoulos G., Avgeropoulou A., Brili S. (2021). Epidemiology and Management of Chronic Thromboembolic Pulmonary Hypertension in Greece. Real-World Data from the Hellenic Pulmonary Hypertension Registry (HOPE). J. Clin. Med..

[25] Karyofyllis P., Demerouti E., Giannakoulas G., Anthi A., Arvanitaki A., Athanassopoulos G., Feloukidis C., Iakovou I., Kostelidou T., Mitrouska I. (2022). Balloon Pulmonary Angioplasty in Patients with Chronic Thromboembolic Pulmonary Hypertension in Greece: Data from the Hellenic Pulmonary Hypertension Registry. J. Clin. Med..

[26] Karyofyllis P., Tsiapras D., Demerouti E., Armenis I., Papadopoulou V., Voudris V. (2022). Sickle cell disease related chronic thromboembolic pulmonary hypertension: Challenging clinical scenario. J. Thromb. Thrombolysis.

[27] Mahesh B., Besser M., Ravaglioli A., Pepke-Zaba J., Martinez G., Klein A., Ng C., Tsui S., Dunning J., Jenkins D.P. (2016). Author Notes Pulmonary endarterectomy is effective and safe in patients with haemoglobinopathies and abnormal red blood cells: The Papworth experience. Eur. J. Cardio-Thorac. Surg..

[28] de Sanctis V., Soliman A.T., Daar S., Tzoulis P., Fiscina B., Kattamis C. (2022). Retrospective observational studies: Lights and shadows for medical writers. Acta Biomed..

[29] Samaga D., Hornung R., Braselmann H., Hess J., Zitzelsberger H., Belka C., Boulesteix A.-L., Unger K. (2020). Single-center versus multi-center data sets for molecular prognostic modeling: A simulation study. Radiat. Oncol..

